# Prehospital Emergency Care in Low- and Middle-Income Countries: A Systematic Review

**DOI:** 10.1017/S1049023X23006088

**Published:** 2023-08

**Authors:** Hari Krishna Bhattarai, Sandesh Bhusal, Francesco Barone-Adesi, Ives Hubloue

**Affiliations:** 1. Program in Global Health, Humanitarian Aid and Disaster Medicine, Università del Piemonte Orientale, Novara, Italy, and Vrije Universiteit Brussel, Brussels, Belgium; 2. Nepal Health Frontiers, Kathmandu, Nepal; 3. CRIMEDIM – Center for Research and Training in Disaster Medicine, Humanitarian Aid and Global Health, Università del Piemonte Orientale, Novara, Italy; 4. Department of Emergency Medicine, Universitair Ziekenhuis Brussel, Brussels, Belgium Research Group on Emergency and Disaster Medicine, Medical School, Vrije Universiteit Brussel, Brussels, Belgium

**Keywords:** EMS system, first responder, LMICs, prehospital emergency, response time, transport care

## Abstract

**Background:**

An under-developed and fragmented prehospital Emergency Medical Services (EMS) system is a major obstacle to the timely care of emergency patients. Insufficient emphasis on prehospital emergency systems in low- and middle-income countries (LMICs) currently causes a substantial number of avoidable deaths from time-sensitive illnesses, highlighting a critical need for improved prehospital emergency care systems. Therefore, this systematic review aimed to assess the prehospital emergency care services across LMICs.

**Methods:**

This systematic review used four electronic databases, namely: PubMed/MEDLINE, CINAHL, EMBASE, and SCOPUS, to search for published reports on prehospital emergency medical care in LMICs. Only peer-reviewed studies published in English language from January 1, 2010 through November 1, 2022 were included in the review. The Newcastle–Ottawa Scale (NOS) and Critical Appraisal Skills Programme (CASP) checklist were used to assess the methodological quality of the included studies. Further, the protocol of this systematic review has been registered on the International Prospective Register of Systematic Reviews (PROSPERO) database (Ref: CRD42022371936) and has been conducted following the Preferred Reporting Items for Systematic Reviews and Meta-Analyses (PRISMA) guidelines.

**Results:**

Of the 4,909 identified studies, a total of 87 studies met the inclusion criteria and were therefore included in the review. Prehospital emergency care structure, transport care, prehospital times, health outcomes, quality of information exchange, and patient satisfaction were the most reported outcomes in the considered studies.

**Conclusions:**

The prehospital care system in LMICs is fragmented and uncoordinated, lacking trained medical personnel and first responders, inadequate basic materials, and substandard infrastructure.

## Introduction

Most of the deaths from trauma, heart attacks, stroke, or any other time-sensitive illnesses occur within the first hour (golden hour) and usually out of the hospital.^1^ Prehospital care is thus a crucial part of emergency medical care and can greatly affect health outcomes.^2^

The importance of prehospital emergencies is often neglected in low- and middle-income countries (LMICs),^3^ and this translates into a substantial toll of avoidable deaths from time-sensitive conditions such as injuries, cardiac problems, and obstetric emergencies.^4^

Not only to traumatic patients, prehospital care is equally essential to obstetric as well as communicable and non-communicable disease patients.^5^ A major proportion of deaths from injuries, especially due to road traffic accidents (RTAs), occurs in LMICs with a large proportion of those deaths occurring before reaching the hospital.^6^ Poor road safety and the lack of appropriate and timely care for injured individuals might be the causes of this high number of deaths.^7^ Care of the injured person due to an accident starts before arrival in the hospital, and it is believed that prompt and efficient prehospital care reduces morbidity and mortality associated with RTAs.^8^ This critical care helps to stabilize patients and prepare them for transport to a health care facility by providing timely and appropriate care in the prehospital setting.^9^ Studies have reported that a significant proportion of deaths and disabilities can be reduced by well-organized prehospital care or Emergency Medical Services (EMS).^10,11^ The key components of EMS, namely notification (time from scene to receipt of call by the dispatch team), activation (time from receipt of call to dispatch), response (time from dispatch to arrival at the scene), on-scene (time from arrival at the scene to departure), and transport (departure from the scene to arrival at the hospital) play a vital role in timely, effective, and integrated care.^12–14^ In LMICs, it is common to witness limited access to health care facilities or trained medical personnel, so prehospital care provided by first responders or other trained emergency medical technicians (EMTs) can make a crucial difference in the outcome of a medical emergency.^15^

The rapid arrival of an ambulance at the scene/patients coupled with trained emergency medical personnel and adequate victim transportation to the hospital may mitigate morbidity, prevent disability, and enhance the survival of patients with time-sensitive illnesses.^16^ As the first point of contact between patients and the emergency care department, dispatching unit personnel not only assess the urgency of a call and dispatch a team accordingly, they also try to give counseling to the caller to minimize the consequences of the emergency and manage the patient/victim.^17^

Quality prehospital emergency care can make an important contribution to reducing avoidable deaths and disabilities, but the public health system has never prioritized emergency medical care, especially in developing countries.^18–20^ The availability of quality prehospital care causes a significant reduction in trauma-related mortality alone.^21^ It is also the foundation for effective disaster response and management of mass-casualty incidents.^22,23^ So, it is a critical component of the health systems and is necessary to improve outcomes of injuries and other time-sensitive illnesses.^24^

To inform the stakeholders for effective policy and program interventions improving the existing prehospital emergency service system, it is essential to gain a deeper understanding of the various domains within the system, such as response time, patient safety, resource utilization, quality of information exchange, and transportation care in resource-poor settings.

There exists a need for a comprehensive assessment of the situation of the prehospital care system in LMICs, as there are few studies conducted in this area. This review can provide insights into the challenges and opportunities for improving emergency care in LMICs.

## Aim

This systematic review aimed to assess the prehospital emergency care system in LMICs with special emphasis on the structure of an EMS system, transport care, prehospital time interval, communication exchange, and patient satisfaction.

## Methods

### Protocol

The protocol for this systematic review has been published in the International Prospective Register of Systematic Reviews (PROSPERO) database (Ref: CRD42022371936) and has been conducted adhering to the Preferred Reporting Items for Systematic Reviews and Meta-Analyses (PRISMA) guidelines (S1 Table; available online only).^25^

### Search Strategy and Selection Criteria

Four electronic databases were systematically searched: PubMed (National Center for Biotechnology Information, National Institutes of Health; Bethesda, Maryland USA), CINAHL (EBSCO Information Services; Ipswich, Massachusetts USA), EMBASE (Elsevier; Amsterdam, Netherlands), and SCOPUS (Elsevier; Amsterdam, Netherlands), for published reports of prehospital emergencies in LMICs using database-tailored search strategy. Boolean logic was used in the databases with search terms including: “pre-hospital emergency,” “Pre-hospital care,” “emergency transport,” and the names of LMICs. A manual search was also performed in the reference lists of the included studies and systematic reviews on similar topics identified in the database search. Studies published from January 1, 2010 through November 1, 2022 were eligible for selection in the review.

The studies retrieved through database search were imported to Zotero citation manager (Version 6.0.26; Corporation for Digital Scholarship; Vienna, Virginia USA). After eliminating duplicate articles in Zotero, reviewers independently performed basic screening (title/abstract) of studies based on the eligibility criteria to proceed to the next step of the screening.

### Criteria for Study Selection

*Inclusion—*Inclusion criteria were as follows:

- Studies assessing the quality or status of prehospital emergency care in at least one of the LMICs based on the World Bank’s (Washington, DC USA) classification.^26^
- Qualitative and quantitative studies published in English language.
- Studies reporting on the six different areas of prehospital emergency care: prehospital emergency care structure, transport care, prehospital times, health outcomes, quality of information exchange, and patient satisfaction were included in this review.

*Exclusion*—Letters to the editor, review articles, and studies published in languages other than English were excluded. Studies that focused on intra-hospital emergency health care or intra-hospital patient transportation were also excluded from the review.

### Data Extraction

Authors individually extracted data from the included studies using a data extraction table developed in Microsoft Excel (Microsoft Corporation; Redmond, Washington USA) for this review. The information extracted from the included studies comprised: (1) author details – name and publication year; (2) study characteristics – study design, geographic location of the study, and sample size (if applicable); and (3) the main findings related to prehospital emergency care.

### Assessment of Risk of Bias

Authors HKB and SB independently assessed the potential risk of bias in the included studies using the Newcastle–Ottawa quality assessment scale (NOS).^27^ This scale assesses the quality of the articles in the domains of selection, comparability, and exposure. The maximum score on the NOS was eight. Studies that scored more than six points were considered of high quality, studies scoring four-to-six points were considered moderate quality, and studies with scores less than four points were considered as being of low methodological quality.^28,29^

The Critical Appraisal Skills Programme (CASP) checklist was used to appraise the health-related qualitative evidence syntheses.^30^ The CASP tool has ten questions across three main areas: internal validity, results, and external validity. Each question on the checklist is scored as either “yes,” “no,” or “cannot tell.”

## Results

### Study Selection

**Figure 1.**
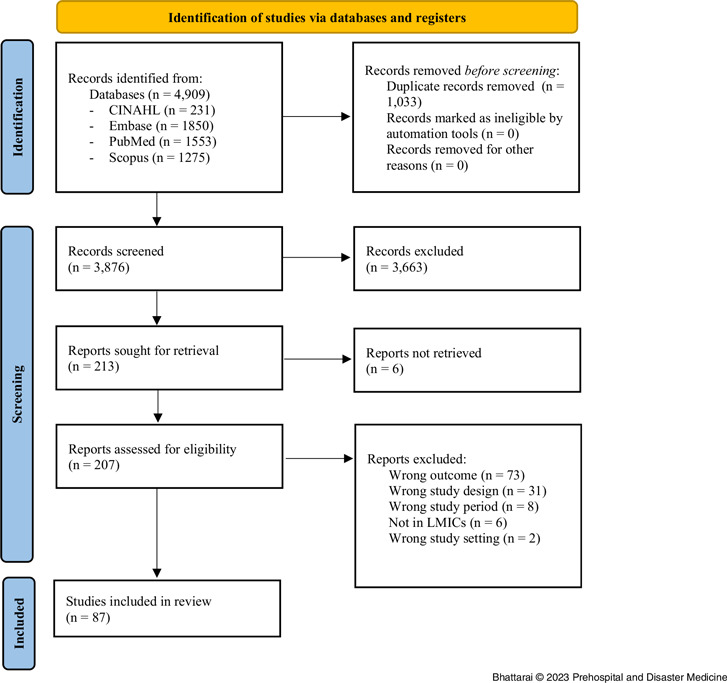
Flow Diagram of the Study Selection. Abbreviation: LMIC, low- and middle-income countries.

The search strategy yielded 4,909 citations from four databases. After duplicate removal, a total of 3,876 studies were retrieved for the title and abstract screening, of which 213 studies were selected for full-text screening. After the full-text screening, 126 studies were excluded for the following reasons: wrong outcome, wrong study design, wrong study period, not conducted in LMICs, full-text not found, and wrong study setting. Therefore, a total of 87 studies met the inclusion criteria and were included in this review, as depicted in Figure 1 using the PRISMA diagram.

### Characteristics of the Included Studies

Of the 87 included studies, the majority were cross-sectional (n = 52) in design, followed by qualitative studies (n = 15), prospective studies (n = 11), and cohort studies (n = 9). Most of the studies were from Iran (n = 23), followed by India (n = 7), South-Africa (n = 7), and Brazil (n = 5). Also, Asian countries represented the maximum number of studies (n = 54), followed by African (n = 23), South American (n = 6), European (n = 3), and North American (n = 1).

The studies included different cases of prehospital emergencies. Overall, among the 87 studies included in the review, trauma and injuries (n = 40) were the major emergency conditions requiring prehospital care. Other studies included emergencies of chronic diseases (n = 21), disasters (n = 7), pediatrics (n = 4), obstetrics (n = 4), and other conditions (n = 11).

The outcomes were categorized into six major categories: prehospital emergency care structure (n = 26 studies), transport care (n = 26 studies), prehospital times (n = 22 studies), health outcomes (n = 18 studies), quality of information exchange (n = 4 studies), and patient satisfaction (n = 3 studies). Some of the studies reported multiple outcomes, therefore, the total number exceeded 87.

### Methodological Quality

The individual scores ranged from three-to-eight for cross-sectional studies on the NOS. Twenty of the studies were classified as being of high methodological quality, 41 were appraised as being of moderate quality, and two were classified as low quality. For the cohort studies, the individual scores ranged from four-to-eight points. Four of the studies were classified as being of high quality, and the remaining five were classified as having moderate methodological quality.

Qualitative studies were subject to quality assessment using the CASP checklist. Out of a total score of ten, eight studies received a score of ten, five studies received a score of nine, and two studies received a score of eight.

### Status of Prehospital Emergency Care Services

*Prehospital Emergency Care Structure—*Twenty-seven studies assessed the prehospital emergency care structure in various countries and regions and identified a range of challenges and deficiencies. Prehospital emergency services provided in most areas were suboptimal. Several studies in South Africa,^31^ Pakistan,^32^ Malawi,^33^ Iran,^34^ Yemen,^35^ and Peru^36^ found uncoordinated, fragmented, and insufficient prehospital care systems. Another study in Iran by Bidgoli, et al showed an unequal distribution of prehospital trauma care facilities between provinces.^37^

On the aspect of human resources, most of the patients were attended by members of the public as first responders.^38–40^ Many studies highlighted a lack of trained medical personnel and first responders, which could lead to delays in providing care and poor outcomes for patients.^34,37,41,42^ Insufficient multidisciplinary teams and poor infrastructure, including road access, lack of basic materials, and uncoordinated and fragmented system, were frequently cited as a challenge to the effective functioning of the prehospital care system.^36,42–45^

Five studies assessed prehospital care and preparedness plans during disasters.^44,46–49^ Issues like lack of a structured disaster management plan, absence of standardized medical teams, shortages of resources, lack of basic knowledge among rescue teams, and ineffective coordination were observed in the studies (Table 1 ^11,31–55^).

**Table 1.** Summary of Findings from Studies Assessing Prehospital Emergency Care Structure

S.N.	Study	Sample and Study Design	Methodological Quality	Country	Type of Emergency	Findings/Conclusion
1	Alinia, et al 2015^41^	18 participants with experience in the field of prehospital services; Qualitative Study	High	Iran	Injuries	Inadequate human resources, poor knowledge about first aid interventions, and lack of organizational coordination were the major challenges.
2	Anest, et al 2016^31^	33 structured interviews with people involved in EMS; Qualitative Study	High	South Africa	Pediatric	Access to the system, infrastructures including road access, policies, and procedure barriers were predominantly around inefficient systems.
3	Bhatti, et al 2013^32^	Managers and ambulance staff; Cross-Sectional Study	Moderate	Pakistan	Injuries	Existence of deficiencies in prehospital care on a selected Pakistani inter-urban road. Training paramedics, arranging essential supplies, and improving formal communication lines between ambulance stations and health facilities are needed.
4	Bidgoli, et al 2011^37^	Cross-Sectional Study	Moderate	Iran	Injuries	Prehospital trauma care facilities were distributed unequally between different provinces that do not reflect the needs in terms of RTM and RTIs.
5	Broccoli, et al 2016^52^	21 FGD with community members and health care providers; Qualitative Study	High	Zambia	Emergency Care Delivery	The prehospital emergency care system needs to strengthen. Substantial reliance on family members and neighbors for transportation, lack of community knowledge, and referral system were major challenges.
6	Chokotho, et al 2017^33^	Focus groups with individuals of first response organizations; Qualitative Study	High	Malawi	Injuries	Access to professional prehospital care in Malawi is almost nonexistent. Community members are not prepared, emergency telephone numbers are unreliable, and almost no ambulances are available to safely transport trauma patients.
7	Djalali, et al 2011^46^	19 interviews with experts and managers responsible for responding to earthquakes; Qualitative Study	High	Iran	Disaster	Absence of a structured disaster plan, absence of standardized medical teams, and shortage of resources.
8	G/Ananya, et al 2021^38^	238 trauma patients; Cross-Sectional Study	Moderate	Ethiopia	Trauma	Relatives and bystanders were the first responders during trauma care. Ambulance utilization for prehospital care was low.
9	Haghparast-Bidgoli, et al 2010^51^	15 prehospital trauma care professionals; Qualitative Study	High	Iran	Trauma	Administration, organization, shortages of professional medical staff, inadequate skills of the current staff, and inappropriate communication were identified as key factors in the inefficient prehospital trauma care process.
10	Khashayar, et al 2010^34^	994 trauma patients; Prospective Study	Moderate	Iran	Trauma	Tehran’s EMS is not capable of providing trauma patients with effective and accurate prehospital care. Lack of up-to-date protocols, training courses, and shortage of ambulances were the main reasons contributing to the ineffectiveness of EMS.
11	Khorasani-Zavareh, et al 2018^42^	18 interviews with EMS personnel; Qualitative Study	High	Iran	Injuries	Prehospital system factors, including the number and location of EMS facilities, type and number of ambulances, and manpower were major barriers to effective prehospital care.
12	Lima, et al 2010^43^	Interviews with managers and health workers at 13 prehospital units; Qualitative Study	High	Brazil	Injuries	Lack of equipment and basic materials, insufficient multidisciplinary teams, and the need for on-going training were observed.
13	Lin, et al 2011^47^	Focus group conducted with the health care workers and survey with patients; Cross-Sectional Study	Moderate	Guatemala	Disaster	It was not prepared to address the community’s health needs after the hurricane as there were no previous plans in place for disaster response for the clinic or the community.
14	Lodhi, et al 2011^48^	83 patients with spinal injuries; Cross-Sectional Study	Moderate	Pakistan	Disaster	Poor prehospital management of spinal injured patients depicts the lack of emergency preparedness as well as the lack of basic knowledge among rescue teams and health care providers about the common trauma management measures.
15	Mawani, et al 2018^53^	187 cardiac patients; Cohort Study	High	Pakistan	Cardiac	There was no survival after a traumatic OHCA in Karachi, Pakistan. There is a strong need to strengthen the prehospital care system and train the general public to deal with emergencies and be able to provide timely bystander CPR.
16	Meena, et al 2018^39^	830 cases of TBI; Prospective Study	Moderate	India	Trauma	Most of the patients were attended by members of the public as first responders but none of them received any transport care.
17	Mohseni, et al 2018^54^	577 traumatic patients; Cross-Sectional Study	Moderate	Iran	Trauma	Prehospital emergency services provided in most of the domains are relatively far from world standards. Immediate measures should be taken by developing standard protocols and training the staff.
18	Mould-Millman, et al 2015^11^	Interviews with over 30 EMS personnel; Cross-Sectional Study	Moderate	Ghana	Emergency Care Delivery	National Ambulance Service in the Ashanti region of Ghana is well poised to meet the regional demand for prehospital emergency care and transport.
19	Mousavi, et al 2022^44^	Interviews with 26 experts in the field of medical emergencies; Qualitative Study	High	Iran	Disaster	Lack of experts, infrastructures, response plans, and organizational coordination were found as challenges in prehospital aerial operations in response to an earthquake.
20	Naser, et al 2022^35^	153 interviews; Qualitative Study	High	Yemen	Emergency Care Delivery	Despite the availability of some formal services, the prehospital care system in Yemen is uncoordinated, fragmented, and insufficient.
21	Nayeri, et al 2021^45^	16 Iranian emergency medical staff; Qualitative Study	High	Iran	Cardiac	Lack of proper organizational structure, facilities, and equipment, and lack of experienced and skilled manpower were the challenges.
22	Ramirez, et al 2014^50^	207 emergency cases; Cross-Sectional Study	Moderate	Uganda	Obstetric	The system in rural Uganda demonstrates that an EMS system is possible, affordable, and highly utilized by communities for life-threatening complaints.
23	Reilly, et al 2019^40^	1,132 TBI patients; Cross-Sectional Study	Moderate	Indonesia, India, Pakistan	Injuries	On-site care was usually provided by a member of the public. The accident victim was rarely accompanied in an equipped ambulance by trained personnel.
24	Sorani, et al 2018^49^	23 experienced individuals in the field of disaster; Qualitative Study	High	Iran	Disaster	Laypeople do not have enough first aid knowledge, communication within the affected area as well as outside the region is usually disrupted, and medical staff and even EMS managers have inadequate knowledge and skills in disasters.
25	Vasa, et al 2021^36^	22 (first responders and community members); Qualitative Study	High	Peru	Emergency Care Delivery	Lack of infrastructure, lack of structured care delivery, unclear protocols, and lack of trust in service providers were barriers to emergency care.
26	Zalihić, et al 2022^55^	1,362 OHCA patients; Cross-Sectional Study	Moderate	Bosnia and Herzegovina	Cardiac	There was an extremely low rate of bystander engagement and no AEDs usage.

Abbreviations: AED, automated external defibrillator; CPR, cardiopulmonary resuscitation; EMS, Emergency Medical Services; FGD, focus group discussion; OHCA, out-of-hospital cardiac arrest; RTM, road traffic mortality; RTI, road traffic injuries; TBI, traumatic brain injury.

*Transport Care*—Twenty-five studies reported a number of issues related to transport care regarding prehospital emergency care. Many studies found that patients were often transported by family members or private vehicles rather than ambulances.^8,10,56–58^ Patients in difficult terrains experienced delays in reaching health facilities. Also, there was a significant association of longer transport time to worse outcomes.^59,60^

Studies have found that the use of automated external defibrillators (AEDs) and Advanced Life Support (ALS) interventions during ambulance transportation can improve patient outcomes.^59,61,62^ However, the percentage of ambulances equipped with AEDs, ventilator, disposable splint, and wheelchair were very far from standards.^57,58,63^

Only a minority of ambulances across LMICs were physician-staffed or had Basic Life Support (BLS)-trained personnel.^64–68^ A study conducted among cardiac emergency cases in Iran found a lower death rate when transported by EMS.^62^ Similarly, a study in Turkey reported a higher short-term mortality rate among pediatric emergencies if the ambulance was staffed by only paramedics.^65^

Overall, factors such as absence of dedicated vehicles, lack of equipment on ambulances, and lack of skilled personnel during transportation were major challenges for effective transport care during an emergency (Table 2 ^8,10,31,56–78^).

**Table 2.** Summary of Findings from Studies Assessing Transport Care

S.N.	Study	Sample and Study Design	Methodological Quality	Country	Type of Emergency	Results
1	Ahidjo, et al 2011^10^	168 patients with SCI; Cohort Study	Moderate	Nigeria	Injuries	The majority were conveyed to the casualty by their relatives and presented after 24 hours of the injury.
2	Anest, et al 2016^31^	33 structured interviews with health care personnel in EMS; Qualitative Study	High	South Africa	Pediatric	The lack of dedicated vehicles for the transportation of pediatric patients was as a barrier to effective prehospital care.
3	Apiratwarakul, et al 2021^69^	271 motorlance operations; Cross-Sectional Study	Moderate	Thailand	Trauma	Almost all of the motorlance operations were found to have no access to AED equipment installed in public areas.
4	Apiratwarakul, et al 2022^61^	891 cardiac emergency cases; Cohort Study	Moderate	Thailand	Cardiac	Motorcycle ambulances equipped with an AED had shorter periods of activation time and response time when compared to traditional ambulances. The use of AEDs increases the number of continuous resuscitations in OHCA patients.
5	Bhat, et al 2021^56^	205 OHCA patients; Cohort Study	Moderate	India	Cardiac	41.5% of patients reached hospital by means other than ambulance. Only 9.8% of patients had received bystander CPR. Only 12.5% of ambulances had BLS-trained personnel.
6	Bhoyar, et al 2020^74^	81 accident victims; Prospective Study	Moderate	India	Injuries	77 (97%) persons who were transported in an ambulance were accompanied by a doctor. Only 19 (23%) accident victims received first aid.
7	Caviglia, et al 2021^73^	28,574 hospital admissions; Retrospective Study	High	Sierra Leone	All Cases	NEMS enhanced the access to hospital care among vulnerable rural populations by overcoming geographical barriers and issue of transport availability.
8	Gonsaga, et al 2012^70^	850 trauma patients; Cross-Sectional Study	Moderate	Brazil	Trauma	Most patients were transported by Urgent Medical Aid Service (SAMU). Fire Brigade (CB) responded more quickly than SAMU, and there was no statistical difference between the services of SAMU and CB in terms of severity of the trauma and mortality rates.
9	Haddadi, et al 2019^63^	500,000 EMS recorded missions; Cross-Sectional Study	Moderate	Iran	Emergency Care Delivery	The percentage of ambulances equipped with AED, ventilator, disposable splint, and wheelchair were very far from standards.
10	Hoang, et al 2021^57^	239 OHCA cases; Cross-Sectional Study	Moderate	Vietnam	Cardiac	EMS transported 20.5% of cases to the hospital with the remaining being transported by private vehicle. No patients received AED before arriving at the hospital.
11	Howard, et al 2014^75^	485 pediatric patients; Cross-Sectional Study	Low	South Africa	Pediatric	AMS remains a safe and viable alternative to non-specialized pediatric transfer and may serve as a potential alternative to specialized pediatric transfer in the Western Cape.
12	Ibrahim, et al 2017^8^	23,537 road traffic injured patients; Cross-Sectional Study	Moderate	Nigeria	Injuries	Only 2.3% of the patients had formal prehospital care and were brought to the hospital by Lagos State Ambulance Service (LASAMBUS). They also had significantly shorter arrival times.
13	Kotwal, et al 2018^76^	10,559 trauma patients; Cross-Sectional Study	Moderate	Afghanistan	Trauma	Most prehospital interventions were provided to patients transported by medical evacuation (MEDEVAC) air. The shortest time for transport but higher mortality was seen with casualty evacuation (CASEVAC) air.
14	Mabry, et al 2012^64^	671 injured patients; Cross-Sectional Study	High	Afghanistan	Injuries	The 48-hour mortality for the critical care-trained flight paramedics (CCFP) was 8% compared to 15% for the standard MEDEVAC.
15	Meghoo, et al 2019^77^	2,029 patients with respiratory distress; Cross-Sectional Study	Moderate	Ukraine	Respiratory	The EMS dispatch center in a medium-sized city in Ukraine has an adequate organizational infrastructure to ensure that a physician-led public ambulance responds rapidly to complaints of respiratory distress.
16	Mowafi, et al 2016^58^	3,498 trauma patients; Prospective Study	Moderate	Zambia	Trauma	Only 5.9 % of the patients were transported by public or private ambulance. The majority did not receive any formal prehospital care.
17	Najafi, et al 2022^62^	2,244 patients with STEMI; Cross-Sectional	Moderate	Iran	Cardiac	The death rate in patients with acute myocardial infarction who used EMS transport was lower than those who used non-EMS transport.
18	Norouzpour, et al 2013^78^	66 gunshot-wound patients; Prospective Study	Moderate	Iran	Trauma	EMS ambulance transport improved patients’ emergency care and standard time intervals were achieved. Upgrade of ambulance equipment and training of private ambulance personnel may be needed as ambulance transportation was not associated with a hospital stay.
19	Oliveira, et al 2022^71^	Cross-Sectional Study	High	Brazil	Cardiac	Reduction in the underlying mortality rate since SAMU implementation. SAMU has the potential to intervene in the prognosis of transported cases.
20	Paravar, et al 2014^59^	2,000 trauma patients; Cross-Sectional Study	High	Iran	Trauma	There was a significant association between longer transport time to worse outcomes and a positive association of survival with ALS interventions.
21	Rosenberg, et al 2020^72^	2,912 motorcycle-related RTCs; Cross-Sectional Study	Moderate	Rwanda	Injuries	SAMU performed interventions for 47% of patients involved in a motorcycle-related RTC. Though injuries occurred frequently, critical trauma cases from motorcycle crashes were uncommon, indicating improved road safety.
22	Sabde, et al 2014^60^	468 parturients; Cross-Sectional Study	Moderate	India	Obstetric	JSY program brought more women into institutions for delivery. Those who experienced delays to reach health facilities were in difficult terrains of the districts.
23	Saz, et al 2021^65^	2,094 critically ill children; Cohort Study	High	Turkey	Pediatric	The short-term mortality rate was higher if the ambulance was staffed by only paramedics.
24	Shrivastava, et al 2014^66^	200 RTA victims; Cross-Sectional Study	Moderate	India	Injuries	Almost 33% of the victims were not aware of the existence of emergency ambulance services. Also, only 7.5% of victims were brought to the hospital in the emergency ambulance, of which only three victims were accompanied by a doctor.
25	Tachfouti, et al 2011^67^	Interviews with persons involved in trauma care; Qualitative Study	High	Morocco	Trauma	At the prehospital care level, only three out of 15 ambulances were equipped with resuscitation equipment and were used rarely. Only one of the ambulance staff out of three was trained in the required skills.
26	Turan, et al 2022^68^	2,094 pediatric patients; Prospective Study	Moderate	Turkey	Pediatric	Only a minority of ambulances were physician-staffed (16.5%), and 72% of the patients were delivered to pediatric emergency departments without notification calls. Mortality occurred in nine patients. If the health care providers were paramedics, they were more likely to avoid mortality by performing any intervention.

Abbreviations: AED, automated external defibrillator; CPR, cardiopulmonary resuscitation; JSY, Janani Suraksha Yojana; SAMU, Urgent Medical Aid Service; SCI, spinal cord injury; STEMI, ST elevation myocardial infarction; RTC, road traffic collision; RTA, road traffic accident; ALS, Advanced Life Support; EMS, Emergency Medical Services.

*Prehospital Time Intervals—*Twenty-two studies assessed prehospital time intervals including activation time, response time, scene time, and transport time. Activation time (Range: 0.4-4.5 minutes), response time (Range: 6.6-24.2 minutes), scene time (Range: 10.3-18.0 minutes), and transport time (Range: 7.2-83.5 minutes) varied widely across the studies and countries.

Studies conducted among trauma patients in India^39^ and Ethiopia^38^ showed that only 34.5% and 56.1%, respectively, were able to reach health facilities within the golden hour. A slightly higher proportion of patients were transported to the emergency centers (ECs) in Rwanda within the golden hour.^79^

Studies have found that the use of specialized vehicles such as motorlances (motorcycles modified to be used as ambulances) and helicopter Emergency Medical Services (HEMS) can lead to shorter response times than traditional ambulances,^61,69,80^ which was in turn associated with improved health outcomes and lower mortality.^59,81,82^ Factors that affected response times included the distance from the hospital, location, type of emergency, and ambulance mechanism. Additionally, several studies found that response times in rural areas were generally longer than those in urban areas^83–85^ (Table 3 ^38,39,59,61,63,69,74,79–93^).

**Table 3.** Summary of Findings from Studies Assessing Prehospital Time Intervals

S.N.	Study	Sample and Study Design	Methodological Quality	Country	Type of Emergency	Results
1	Apiratwarakul, et al 2021^69^	271 motorlance operations; Cross-Sectional Study	Moderate	Thailand	Trauma	The activation time and response time of motorlances were shorter than a conventional ambulance.
2	Apiratwarakul, et al 2022^61^	901 cases of EMS operation; Cohort Study	Moderate	Thailand	Cardiac	Activation time: 0.44 minutes (motorlance) versus 1.42 minutes (traditional ambulance). Response time: 7.2 minutes (motorlance) versus 9.25 minutes (traditional ambulance).
3	Aziz, et al 2020^86^	82 OHCA cases; Cohort Study	High	Malaysia	Cardiac	The mean ambulance response time was 14.91 minutes.
4	Bayiga, et al 2019^87^	74 road traffic crash victims; Cross-Sectional Study	Moderate	Uganda	Injuries	Prehospital care time ranged between 10 and 220 minutes. Mean activation time: 4.58 minutes. Scene to hospital transport time (mean): 19.07 minutes.
5	Bhoyar, et al 2020^74^	81 accident victims; Prospective Study	Moderate	India	Injuries	The average time for victims to reach the hospital was 38.1 minutes.
6	Cardoso, et al 2014^88^	220 rescue operations; Prospective Study	Moderate	Brazil	Trauma	Average prehospital time: 42 minutes. Average response time: 10 minutes.
7	Caviglia, et al 2021^81^	6,387 obstetric emergencies; Cross-Sectional Study	High	Sierra Leone	Obstetric	The proportion of emergency obstetric referrals with a prehospital time within 2 hours was 58.5% during the rainy season and 61.4% during the dry season. There is a clear association between increasing prehospital time and maternal and perinatal mortality.
8	Di, et al 2020^83^	300 emergency cases; Cross-Sectional	High	Malaysia	All Cases	84.7% of the cases were determined to have delayed ambulance response time. The ambulance response time is 14 minutes. Delayed ambulance response time was associated with distance from the hospital, location, type of emergency, and ambulance mechanism.
9	G/Ananya, et al 2021^38^	238 trauma patients; Cross-Sectional	Moderate	Ethiopia	Trauma	Only one-half of the patients presented to the health facility within the golden hour.
10	Ghadimi, et al 2021^89^	204 AIS patients; Cross-Sectional Study	High	Iran	Cardiac	The delay in deciding to contact the emergency service or making the effort to refer to medical centers (204.74 minutes) was longer compared to the time of patient transfer to the hospital (83.52 minutes).
11	Ghaffarzad, et al 2021^80^	268 emergency cases; Cross-Sectional Study	Low	Azerbaijan	Trauma	The mean transfer time was 54.68 (SD= 14.17) minutes, while the mean estimated ground route time was 86.38 (SD = 26.26) minutes. HEMS missions have reduced patient transport time and also made mortality rate closer to international standards.
12	Haddadi, et al 2019^63^	500,000 EMS missions; Cross-Sectional Study	Moderate	Iran	All Cases	The mean response time, scene time, and transport time to the hospital were 15.00 (SD = 10.88), 18 (SD = 11.48), and 15.00 (SD = 11.20) minutes, respectively. All were more than standard time.
13	Khanizade, et al 2021^90^	2,659 heart attack patients; Cross-Sectional Study	Moderate	Iran	Cardiac	The average of activation, response, on-scene, transportation, recovery, and total time intervals were 3:30, 7:56, 15:15, 13:34, 11:07, 12:11, and 41:25, respectively.
14	Mahama, et al 2018^91^	652 trauma cases; Cross-Sectional Study	High	Ghana	Trauma	The average response time to patients was 16.9 (SD = 0.7) minutes and the median transportation time of the patient was 82 minutes.
15	Mbanjumucyo, et al 2016^79^	1,668 trauma patients; Cohort Study	Moderate	Rwanda	Trauma	Median transport time was 32 minutes. Overall, 82.7% of patients were transported to the EC in less than one hour.
16	Meena, et al 2018^39^	830 cases of TBI; Prospective Study	Moderate	India	Trauma	Time duration to reach definitive treatment centers was <1 hour in 34.58%.
17	Mohammadi, et al 2014^92^	500 emergency cases; Cross-Sectional Study	Moderate	Iran	All Cases	The mean interval between receiving the mission to reaching the scene, between reaching the scene to moving from the scene, and between moving from the scene to a health center was 7.28, 16.73, and 7.28 minutes.
18	Nadarajan, et al 2021^84^	525 cases of heat illness; Cross-Sectional Study	Moderate	India	Disaster	Time (mean): call to scene-24:23, scene duration-10:38, scene to hospital-26:38. The highest incidence of calls came from rural areas, however, the time to respond in rural areas was longer than that in urban areas.
19	Paravar, et al 2013^85^	1,600 RTA cases; Cross-Sectional	Moderate	Iran	Injuries	The mean prehospital time intervals (minutes); response, scene, and transport for all patients 6.6 (SD = 3.1), 10.7 (SD = 5), and 13 (SD = 9.8), respectively. Time intervals on roads out of the city were higher than those on city streets.
20	Paravar, et al 2014^59^	2,000 trauma patients; Cross-Sectional Study	High	Iran	Trauma	The mean response time, at scene time, and transport time were 6.6 (SD = 3), 11.1 (SD = 5.2), and 12.8 (SD = 9.4), respectively. There was a significant association of longer transport time to worse outcomes.
21	Sladjana, et al 2011^82^	591 OHCA patients; Prospective Study	High	Serbia	Cardiac	The median time of recognition OHCA was 5.5 minutes, call receipt was one minute, and the call-response interval was seven minutes. The emergency response time within four minutes was associated with improved survival.
22	Zimmerman, et al 2020^93^	3,209 TBI patients; Cross-Sectional Study	High	Tanzania	Trauma	The most common wait time from injury occurrence to hospital arrival was 1.1 to 4.0 hours (31.9%). No significant associations between time to arrival and in-hospital outcome.

Abbreviations: AIS, acute ischemic stroke; EC, emergency center; OHCA, out-of-hospital cardiac arrest; TBI, traumatic brain injury; RTA, road traffic accident; EMS, Emergency Medical Services; HEMS, helicopter Emergency Medical Services.

*Health Outcomes—*Eighteen studies assessed health outcomes following prehospital emergency care. The studies reported that prehospital care interventions performed were associated with EMS personnel’s skills and educational level. Prehospital systems of trained paramedics and layperson first responders reduced trauma mortality in severe RTA injuries.^94,95^ Increasing prehospital time was associated with adverse outcomes and mortality among emergency cases.^81^ Especially following cardiac emergencies like out-of-hospital cardiac arrest (OHCA), the survival rate was low. Factors like bystander cardiopulmonary resuscitation (CPR), public availability of AED, and public awareness of early cardiac arrest were more likely to increase the survival rate among cardiac emergencies and RTAs.^86,96–98^ Advanced transport systems like HEMS and Urgent Medical Aid Service (SAMU) have reduced transportation time and played a crucial role in reducing mortality.^80,99,100^ A study conducted by Sobuwa, et al^101^ showed that prehospital intubations performed among traumatic brain injury patients did not demonstrate improved outcomes, however, another study conducted in South Africa reported a 98% success rate of prehospital endotracheal intubation^102^ (Table 4 ^80,81,86,94–108^).

**Table 4.** Summary of Findings from Studies Assessing Health Outcomes

S.N.	Study	Sample and Study Design	Methodological Quality	Country	Type of Emergency	Results
1	Adib-Hajbaghery, et al 2014^94^	400 patients with multiple trauma; Cross-Sectional	Moderate	Iran	Trauma	The quality of spine and limb immobilizations was undesirable in more than 90% of cases. A significant association was observed between the quality of spine and limb immobilization and the EMS workers’ education level.
2	Aziz, et al 2020^86^	82 OHCA cases; Cohort Study	High	Malaysia	Cardiac	The survival rate to admission was 12.2%, while the survival rate to discharge was 1.2%. Improvement in response time, public availability of AED, and public awareness of early cardiac arrest and CPR are required to increase survivability.
3	Booley, et al 2015^103^	110 patients with symptomatic hypoglycemia; Cross-Sectional	Moderate	South Africa	Chronic Disease	More than one-half of patients who received prehospital treatment and discharge for SH had recurrent symptoms post-reversal by EMS staff.
4	Caviglia, et al 2021^81^	6387 obstetric emergencies; Cross-Sectional	High	Sierra Leone	Obstetric	There is a clear association between increasing prehospital time and maternal and perinatal mortality.
5	Chen, et al 2021^96^	25,421 cases of cardiac arrest; Prospective Study	High	China	Cardiac	The survival rate after OHCA was low. Bystander CPR was indirectly associated with an 8.0% increase in survival rate.
6	Dharap, et al 2017^104^	1,181 trauma patients; Prospective Study	High	India	Trauma	Those who are directly taken to tertiary care trauma centers have a significantly better chance of survival than those transferred from other hospitals, probably because of deficient initial care.
7	El-Sayed, et al 2017^97^	271 patients with OHCA; Cross-Sectional	High	Lebanon	Cardiac	Prehospital CPR was done by EMS for 43.2% of the patients. Survival of EMS-treated OHCA victims in Lebanon is not as expected.
8	Ghaffarzad, et al 2021^80^	268 emergency cases; Cross-Sectional	Low	Azerbaijan	Trauma (Major)	HEMS missions have reduced patient transport time and also made the mortality rate closer to international standards.
9	Luz, et al 2010^99^	>5,000 cardiac emergencies; Cross-Sectional	Moderate	Brazil	Cardiac	The presence of Urgent Medical Aid Service (SAMU) was significantly associated with indicators of stroke and AMI mortality.
10	Murad, et al 2012^95^	128 in the treatment group and 77 in the control group; Cohort Study	High	Iraq	Injuries	A two-tier prehospital system of trained paramedics and layperson first responders reduces trauma mortality in severe RTA injuries.
11	Niekerk, et al 2018^100^	204 HEMS cases; Cross-Sectional	Moderate	South Africa	Trauma (Major)	The clinical interventions performed by helicopter crews tend to have a positive effect on patient stability.
12	Raffee, et al 2017^98^	79 OHCA and 257 IHCA cases; Cross-Sectional Study	High	Jordan	Cardiac	The overall survival rate for OHCA was 2.97%. The survival rate increased to 4.3% if CPR was performed before arriving at the hospital. Only 22% of the OHCA cases had CPR performed mainly due to a lack of knowledge and skills of bystanders.
13	Schauer, et al 2018^105^	19,485 male and 533 female casualties; Cross-Sectional	High	Iraq and Afghanistan	Injuries	No difference in survival between males and females following prehospital combat casualty care.
14	Schauer, et al 2018^106^	802 pediatric cases; Cross-Sectional	High	Iraq and Afghanistan	Trauma	Pediatric trauma subjects intubated in the prehospital setting had higher injury severity scores and low survival rates.
15	Sobuwa, et al 2013^101^	124 TBI patients; Cohort Study	Moderate	South Africa	Trauma	Patients who underwent basic airway management had a higher proportion of a good outcome (72.9%) than patients who were intubated in the prehospital setting.
16	Stassen, et al 2018^102^	48 HEMS cases; Cross-Sectional	Moderate	South Africa	Trauma	The first pass success rate of intubation was 79% with an overall success rate of 98%. In LMICs where hospitals are often remote or poorly accessible, prehospital endotracheal intubation might be of value.
17	Wang, et al 2022^107^	1533 OHCA patients; Prospective Study	Moderate	China	Cardiac	Prehospital advanced airway management (AAM) and the combined treatment of AAM and adrenaline in OHCA patients are both associated with an increased rate of ROSC.
18	Wylie, et al 2022^108^	926 ETI cases; Cross-Sectional	High	South Africa	All Cases	Non-physician performed FPS rate was 75.3%, with an overall success rate of 95.7%.

Abbreviations: AED, automated external defibrillator; AMI, acute myocardial infarction; CPR, cardiopulmonary resuscitation; EMS, Emergency Medical Services; ETI, endotracheal intubation; HEMS, helicopter Emergency Medical Services; IHCA, in-hospital cardiac arrest; LMIC, low- and middle-income countries; OHCA, out-of-hospital cardiac arrest; ROSC, return of spontaneous circulation; SH, symptomatic hypoglycemia.

*Patient Satisfaction—*Three studies reported on the satisfaction of patients regarding prehospital care and all were conducted in Iran.^109–111^ Aboosalehi, et al^109^ found that almost 80% of emergency patients were highly satisfied with the services provided by Tehran EMS. Also, high educational and economic status, proper sent vehicle, and accurate diagnosis were amongst the factors leading to higher satisfaction among patients.^109^ Another study found that patient satisfaction with the dispatcher was good, but satisfaction level with EMTs’ performance, physical situation, and facilities inside the ambulance was moderate^110,111^ (Table 5 ^109–111^).

**Table 5.** Summary of Findings from Studies Assessing Patient Satisfaction

S.N.	Study	Sample and Study Design	Methodological Quality	Country	Type of Emergency	Results
1	Aboosalehi, et al 2022^109^	1,100 emergency cases; Cross-Sectional Study	High	Iran	All Type	Almost 80% of the participants were highly satisfied with the services provided by Tehran EMS. Patients with high educational status, high economic status, proper sent vehicle, and accurate diagnosis showed higher satisfaction.
2	Heydari, et al 2017^110^	450 emergency cases; Cross-Sectional Study	Moderate	Iran	All Type	Patient satisfaction with the dispatcher was good, and satisfaction level with the technicians’ performance, physical situation, and facilities inside the ambulance were moderate.
3	Maghaminejad, et al 2016^111^	400 multiple trauma cases; Cross-Sectional Study	Moderate	Iran	Cardiac	The quality of prehospital circulatory management provided to patients with multiple traumas was unfavorable. A significant relationship was observed between the quality of circulatory management and type of trauma and staff’s employment status.

Abbreviation: EMS, Emergency Medical Services.

*Quality of Information Exchange—*Four studies assessed the quality of information exchange in the prehospital setting. It appeared that the quality of information exchange in the prehospital setting was an issue that needed to be addressed. Communication barriers between dispatch personnel and medical facilities/EMS personnel were deemed to be a high priority.^31^ The dispatching unit personnel in prehospital emergency care were confronted with various interactional, organizational, and professional issues.^112^ Also, inter-facility communication was found as poor in a study conducted in Ethiopia^113^ (Table 6 ^31,112–114^).

**Table 6.** Summary of Findings from Studies Assessing the Quality of Information Exchange

S.N.	Study	Sample and Study Design	Methodological Quality	Country	Type of Emergency	Results
1	Abebe, et al 2018^113^	662 RTC patients; Cross-Sectional Study	High	Ethiopia	Injuries	Among referred patients, inter-facility communication was poor (57.7%). Inter-facility referral appears a primary contributor to low-acuity ambulance use.
2	Anest, et al 2016^31^	33 structured interviews with health care personnel in EMS; Qualitative Study	High	South Africa	Pediatric	In a single-center middle-income setting, communication barriers between dispatch personnel and medical facilities/EMS personnel were deemed to be a high-priority intervention to improve prehospital care delivery.
3	Mohammadi, et al 2022^112^	18 interviews with dispatch personnel; Qualitative Study	High	Iran	All Type	The dispatching unit personnel in prehospital emergency care are confronted with various interactional, organizational, and professional issues.
4	Mucunguzi, et al 2014^114^	Cross-Sectional Study	Moderate	Uganda	Obstetric	Reliable communication and transport services increased access to and utilization of maternal health services, particularly cesarean delivery services.

Abbreviations: EMS, Emergency Medical Services; RTC, road traffic collision.

## Discussion

This systematic review assessed the prehospital emergency care services across LMICs. Different areas of prehospital care including the structure of a system, transport care, prehospital time interval, communication exchange, and patient satisfaction were explored.

Most of the studies across LMICs reported the absence of a structured system for prehospital emergency care. For example, studies in South Africa,^31^ Pakistan,^32^ Yemen,^35^ Iran,^34,35^ and Peru^36^ found uncoordinated and fragmented systems without proper protocols. Also, poor access, lack of infrastructure, lack of experienced and skilled EMS personnel, and poor communication, were among the major reasons behind ineffective prehospital care delivery in developing countries.^31,36,37,41,43–45^ In a few cases, the prehospital care system was well poised to meet the demand for prehospital emergency care and transport. Mould-Millman, et al^11^ found that the National Ambulance Service (NAS) in a region of Ghana was well poised to meet the demand for prehospital emergency care and transport.^11^ Similarly, a study in rural Uganda demonstrated an affordable and highly utilized, newly implemented EMS system.^50^ Prehospital care may be neglected and less prioritized in LMICs.^115^ These countries often have limited financial resources to invest in emergency care systems, which can lead to shortages of equipment, ambulances, and trained personnel. Additionally, developing countries nowadays are facing the double burden of communicable diseases and chronic diseases, so they may have to focus on primary health care and disease prevention rather than implementing and strengthening the EMS system.^116,117^ The recent coronavirus disease 2019/COVID-19 pandemic also had an impact on the structure and functioning of the EMS systems. One of the main impacts has been the need to prioritize infection control measures to contain the virus which has resulted in changes to the way EMS operates, ultimately affecting the ability of EMS personnel to quickly and safely transport patients to hospitals.^118,119^

Survival from severe injuries and time-sensitive illnesses is linked to the rapid initiation of treatment. This goal is achieved when a system of prehospital transport – formal or informal – is available to transport patients in the safest and fastest possible way to the nearest ECs.^58,120^ Unfortunately, this essential component of effective emergency care is lacking in many LMICs; as a result, 90% of injury deaths occur in these countries.^121^

A shortage of trained emergency personnel is another impediment to establishing a prehospital emergency response system. Working as an emergency technician is one of the most stressful jobs, and the lack of additional compensation and incentives might have forced them to alter their job or attract newer ones to the job.^122^ In resource-poor countries, involving and providing training to the community members and lay responders might have a meaningful impact on the emergency service.^51^ Having trained paramedics or physicians with knowledge of BLS in an ambulance is important for prehospital care as it allows for prompt and appropriate treatment of patients in emergencies.^123^ Trained personnel can provide life-saving interventions such as CPR, the use of an AEDs, and airway management, all of which can greatly improve a patient’s chances of survival and recovery.^124,125^ Studies in India,^66^ Zambia,^58^ and Vietnam^57^ showed only 7.5%, 5.9%, and 20.0% of emergency patients were transported by public or private ambulances. Also, ambulances were often lacking life-saving interventions^57,63,69^ and BLS-trained personnel.^56,66–68^

Efficient time management is one of the key mechanisms to reducing mortality in emergency patients, especially for trauma and injuries. It is widely accepted that if injured patients do not receive definitive care within the first 60 minutes, the golden hour, of injuries, the chance of mortality significantly increases.^126,127^ A very low proportion of trauma patients in India and Ethiopia were transported to the nearest EC within the golden hour.^38,39^ Prehospital partial time intervals such as response time, scene time, and transport time varied across the LMICs, which might have been greatly influenced by factors distinct from systems of EMS, such as traffic congestion or geographic factors that impede rapid transport.

Motorlances in Thailand showed shorter activation and response time compared to conventional ambulances.^69^ Due to its small structure, it can easily pass through narrow passages/roads, as well as being able to pass through gridlock traffic in confined areas.^128^ Similarly, patients transported by Lagos State Ambulance Service (LASAMBUS) in Nigeria also had shorter arrival times.^8^ The SAMU that dispatches a team of emergency medical personnel to the scene to provide on-site medical care and transports the patient to a hospital for further treatment was seen across different countries. Studies in Brazil^70,71^ and Rwanda^72^ showed a reduction in the underlying mortality rate since SAMU implementation. Similarly, National Emergency Medical Service (NEMS) in Sierra Leone enhanced access to hospital care among vulnerable rural populations by overcoming existing barriers such as geographical accessibility and transport availability.^73^

Not having sufficient funds to purchase expensive medical equipment or train EMTs, inadequate regulations and lack of oversight, and poor communication or collaboration between different providers could be the major challenges to effective transport care in LMICs. Also, difficult terrain or geography and challenging weather can greatly affect prehospital transport, which is evident in the findings of studies conducted in Sierra Leone,^81^ Iran,^85^ and India.^84^

Most of the studies suggested that the health outcome following prehospital care in LMICs is generally poor. Survival following cardiac emergencies like OHCA and severe trauma was low.^96–98^ Some studies found improved outcomes when patients were provided with BLS measures and airway management in the prehospital setting.^96,101^

Longer response time, unavailability of BLS measures like AED and CPR, and unskilled EMS personnel are the reasons for poor health outcomes following prehospital emergencies. Additionally, cultural and societal factors, such as lack of education about emergency care and limited trust in the health care systems, can also contribute to poor outcomes.^36,52^

As prehospital care is provided outside of the hospital, a focus on both the administrative and programmatic aspects of health care delivery is required, which demands strong political commitment.^129^ Poor commitment by decision makers at all levels of management is a repeatedly mentioned barrier to effective care delivery.^130^

As there are many challenges to the prehospital system in the LMICs, there are also possible opportunities for improvement. Collaboration between different stakeholders, including governmental agencies, health care providers, civil society organizations, and international organizations, can leverage expertise, resources, and networks to improve prehospital care in poor-resource settings. Many studies have mentioned the significant impact of community members and lay responders in prehospital care delivery. Engaging and empowering communities can increase the demand for prehospital care and support efforts to improve the quality and accessibility of services. Also, military teams can be implemented in the prehospital system, especially with their participation in the airlifting of casualties and the provision of necessary resources.^46^

## Limitations

This systematic review has certain limitations. There might be some improvements in the EMS during the study period (January 1, 2010 through November 1, 2022), and thus these situations might have changed. As the results were not retrieved from all LMICs, the findings are limited to those nations for which references were retrieved. Moreover, only English and peer-reviewed articles were sought, and the gray literature was not taken into consideration. The inclusion criteria were broad, which could have led to the inclusion of heterogeneous outcomes.

Despite a few limitations, this study made a comprehensive assessment of different domains of prehospital care in LMICs. A range of challenges and barriers were identified in the system, which could be advantageous in designing and implementing policies for the proper functioning and strengthening of the prehospital system in LMICs.

## Conclusion

The implementation and situation of a prehospital emergency care system varied across LMICs. Overall, most LMICs lack an organized prehospital care system and are relatively far from the acceptable standard. Further, the lack of trained medical personnel and first responders, poor infrastructure, lack of basic materials, and inadequate transport care are the key challenges.

Overall, stakeholders should focus on developing and implementing emergency care guidelines and protocols that are tailored to the specific needs of their countries. In addition to this, increasing resources for emergency care, investing in training for EMS personnel, improving infrastructure, and establishing a coordinated system for emergency care to improve communication and coordination should be the priority for concerned stakeholders.

## Supplementary material

For supplementary material accompanying this paper visit https://doi.org/10.1017/S1049023X23006088. click here to view supplementary material
